# Unleading during a pandemic: Scrutinising leadership and its impact in a state of exception

**DOI:** 10.1177/17427150211063382

**Published:** 2022-04

**Authors:** Selen Kars-Unluoglu, Carol Jarvis, Hugo Gaggiotti

**Affiliations:** Bristol Business School, 1981 University of the West of England, Bristol, UK

**Keywords:** state of exception, control, COVID-19, critical leadership studies, unleading, media analysis

## Abstract

Characterising COVID-19 pandemic as a ‘state of exception’, we might expect great hero models of leadership to come to the fore. Instead, drawing on a thematic analysis of 246 news articles, this paper illustrates something different: communities, companies, individuals picked-up the leadership mantle but were reluctant to frame their practices under a leadership rhetoric. The paper explores spontaneous initiatives and leaderly actions that were made visible during the pandemic and proposes practice-based implications for redrawing leadership conceptualisations. These practices, coined as unleading, are characterised under four dimensions: unconditionality and social intention; purposeful action in the absence of an achievement motivation; sensing and attending to local conditions; and confident connecting and collaborating. The analysis and discussion of the four dimensions affirm that while leading and unleading are always present when organising, they are more or less visible and practiced depending on organisational, social and individual circumstances. The paper concludes by surfacing questions and reflections for the future of unleading and implications for leadership theorising and practice.

## Introduction

How the political and economic class have reacted in seeking control of the exceptional circumstances of the COVID-19 pandemic has framed many of the discussions of what Wilson (2020) called pandemic leadership. However, the inspiration for this paper emerged from a different intellectual inquiry. As scholars living and working during the pandemic, we were struck by the volume, proactivity and resourcefulness of initiatives reported from unexpected places, and their contrast with the response from many in formal leadership positions, including those in government and leading corporations.

Mainstream rhetoric suggests that exceptional circumstances impose strong leadership practices, with the assumption that leadership is the product of individual, permanent leaders’ actions; therefore, an absence of ‘successful leaders’ personas, thoughts and actions’ (Collinson, 2005: 1423) implies an absence of leadership. However, the pandemic surfaced not only leaders’ expected interventions, but also the blossoming of spontaneous actions and uncontrolled initiatives, what Raelin (2011) describes as leaderful practices, which go unrecognised through the mainstream lens.

Our objective in this paper is to make visible these often-overlooked actions to capture their conceptual richness. From this, we can begin to rethink and reimagine how we lead in organisations. Drawing on qualitative thematic analysis of news articles published on leaderly actions and initiatives, that in more usual times remain invisible, we propose the concept of *unleading*. The choice of a verb, instead of a noun, is a purposeful attempt to unhook leading/unleading from the extraordinariness of individual actors – the ‘who’ – and to focus attention on what has emerged in the day-to-day and the ‘how’ (Raelin, 2016). Mindful of Alvesson and Sveningsson’s (2003) warning that the act of naming someone a (un)leader may convert their unremarkable acts to be seen as remarkable in their and others’ eyes, we shy away from the noun form. We also see a connection between *un*leading and thriving in *un*certainty, *un*predictability, and the *un*known. Aligned with the use of these words in the literature as spaces for creative disruption, generative action, and innovation, we suggest unleading may help people craft alternative productive ways to exist in organisations.

The findings suggest four dimensions to unleading: unconditionality and social intention; purposeful action in the absence of an achievement motivation; sensing and attending to local conditions; and confident connecting and collaborating. Building on complexity theory, notably Complex Responsive Processes of Relating (Stacey, 2000, 2010), we show how the elements constituting these dimensions intertwine and facilitate meaningful action in the face of deep uncertainty and unpredictability.

In making practices of unleading visible, we see them emerging from everywhere and nowhere, and not always as a contested alternative to leadership or as a response to lack of leadership. As such, we contend that leading and unleading are not binary opposites, but both are always present when organising, being more or less visible and practiced depending on organisational, social and individual circumstances. In this sense, we found inspiration in many critical leadership colleagues who have already given thought to these questions, though perhaps not framed in the terms we decided to use.

In what follows, we first review the leadership literature and its complications followed by an analysis of how the pandemic could be considered a ‘state of exception’ calling for ‘strong leadership’, using Government Press Briefings (2020) to frame our discussion. We follow with a justification of discourse analytical research as our methodological choice for making visible the spontaneous actions that emerged during the pandemic. We then proceed to our analysis of the dimensions of unleading and their elements to explain the practices underpinning these actions. We finish by surfacing questions and reflections for future research and implications for leadership practice.

## Theoretical background

Leadership has long been conflated with attributes such as being decisive, assertive, strong and brave. Leaders who do not display these traits are seen to lack gravitas and disregarded as weak willed (Blake-Beard et al., 2020). Whilst over the past decades, the scholarly and practitioner debate has included previously excluded attributes, such as compassion and sympathy (Adler and Osland, 2016; Gerzema and D’Antonio, 2013), the traditional view of how a leader should act, still shapes people’s expectations of a leader, particularly in times of crisis.

The pandemic has amplified the problematics of this traditional view and its performance in leaders’ reactions. Most in evidence is the putative totalitarian tendency to monopolise actions and decisions in every instance, to act as ‘guardians of themes’ (Bate et al., 2004: 62) and take control. World leaders like Bolsonaro, Trump, Johnson and Modi combined the tough and forceful macho deploying war-like metaphors (Branicki, 2020) common in crisis management (Branicki et al., 2019). With some notable exceptions, like Jacinda Ardern, who spoke not of war against enemies but of working together with kindness, the hegemonic discourse rested on the assumption that the pandemic can be solved through a public display of strength and bravery (Alcadipani, 2020). This ‘ideal of the warrior that does not fear the virus’ (Alcadipani, 2020: 739) and its constant construction and performance proved problematic, exposing organisations and societies to danger. Yet, despite the apparent failure of these leadership performances in guiding us through the pandemic, leaders continued to re-entrench the logic of control and power supporting them. Agamben’s (2005) interpretation of the state of exception helps us understand why this has been the case.

### A pandemic as a state of exception

The pandemic can be described as what Agamben (2005) terms a ‘state of exception’. Reflecting on the national ethos of France, Switzerland, Germany, Italy, England and the US, Agamben argues that the ideal of the perfect nation-state has inspired most authoritarian leadership exercises. Indeed, ‘between 1934 and 1948, in the face of the collapse of Europe’s democracies, the theory of the state of exception saw a moment of particular fortune’ (Agamben, 2005: 6). Gaggiotti and Marre (2017) posit that the conceptualisation and consolidation of the theory of the state of exception during this period permeated most of the public and organisational understandings of leadership, exacerbating aggressive, competitive and performance-driven leadership performances.

Drawing on Agamben’s ideas, we argue that in normal circumstances, leaders consider organisations to be in a ‘state of potential perfection’. Unexpected circumstances, when they arise, are interpreted by leaders as ‘states of exception’, threatening and undermining their organisations with the menace of ‘permanent imperfection’. Agamben describes how these menaces are characterised as ‘impurities’ – threats to the ideal of attaining complete perfection. Leaders must protect their organisations, at any cost, from these menaces.

According to Agamben, a ‘state of perfection’ can only be achieved through the exercise of calculated, rationalised leadership control, avoiding any sort of spontaneity. This demands those in leadership roles *tell* the community their responsibilities, mobilise and command to face things that must be faced (like social distancing and quarantine), and coerce followers into line to establish stability and normalcy by any means necessary (Grint, 2020).

According to Agamben, the leader’s authority to call a state of exception is the perfect opportunity for leaders to justify simplistic interventions to take control by fabricating enemies and justifying public acts of evil against those enemies. Since wars are typical contexts to justify leaders’ exceptional *modus operandi*, perhaps unsurprisingly, the pandemic was framed as a war-like ‘state of exception’. While some leaders, like Trump and Modi, have used the pandemic to fabricate visible enemies like China (Santis, 2020) and Muslims (Prasad, 2020), many more leaders waged a war against an invisible enemy – a virus. As Masu (2020) writes, ‘the war-time imagery is always compelling. It identifies an enemy (the virus), a strategy (flatten the curve), the front-line warriors (health-care personnel), the home-front (people isolating at home), and the traitors and deserters (people breaking the social-distancing rules)’. The war-like imagery was then used by leaders to justify exceptional practices and a rhetoric of full control. This control starts with the labelling of a crisis as ‘a state of exception’ to employ a claim of urgency (Spector, 2020), and framing the state of exception in terms of ‘the people’ versus those responsible for it (Tourish, 2020). The stronger the control logic the leader calls on, the more surveillance is employed to assure compliance (Tourish, 2020). And the power reinforced and extended during the state of exception may not disappear when the contingencies supporting it (i.e. the pandemic) end (Agamben, 2020).

UK Government Ministers’ press briefings on COVID-19, from the first on 3^rd^ March to 26^th^ April 2020, illustrated how this centralising control logic of the state of exception has played out, with war-time metaphors abounding. Suppliers were ‘responding to our call to arms’ (April 10^th^, *Hancock*) and health, care and other key workers were the heroes ‘on the frontline in this battle’ (April 15^th^, *Hancock*). The Government ‘put aside ideology and orthodoxy to mobilise the full power and resources of the British state’ (March 26^th^, *Sunak*), initially focussing on large infrastructure projects, from Nightingale hospitals, to building centralised testing laboratories, whilst the military were enlisted to provide logistical support ‘establishing strategic co-ordination centres across the whole country...led by gold commanders’ *(*March 29^th^, *Jenrick*). But perhaps war-time leadership in a state of exception was best encapsulated in the Prime Minister’s speech on the evening the UK was placed in lockdown:

But in this ***fight*** we can be in no doubt that each and every one of us is directly ***enlisted***. Each and every one of us is now ***obliged*** to join together … And we will ***come through it stronger*** than ever. We will ***beat*** the coronavirus and we will ***beat it together***. And therefore, I urge you at this moment of national emergency to ***stay at home, protect our NHS and save lives***! (March 23^rd^, *Johnson,* emphasis added)

There is no denying that this rhetorical style partly captured the cognitive and emotional aspects of the unexpected transformation the population experienced. ‘Food service, grocery and warehouse workers might not have thought about their jobs as dangerous prior to their occupations becoming deemed essential work’ (Gardiner and Fulfer, 2020: 3). However, these rhetorical tactics fell short in leading through a pandemic shown to be more dispersed and multifaceted than war, and without the characteristics of a single and permanent enemy to defeat (Grint, 2020). The symbolic manipulation of the pandemic at the discursive level has been to the detriment of public leadership causing an evaporation of institutional trust (Dzhurova, 2020), whilst its characterisation as a state of exception impeded the emergence of alternative leadership discourses other than the authoritarian.

### From leadership to unleading

The failure of these leadership performances in guiding us through the pandemic offered the opportunity to disrupt the rhetoric that justifies the monopolisation of decisions and actions during states of exception by those who are in leadership roles. Other scholars (e.g. Bahn et al., 2020; Cherneski, 2020; Wilson, 2020) have also highlighted ways in which the pandemic questioned and broadened traditional conceptualisations framing ‘strong’ leadership and challenged the hegemonic masculine representations.

However, unlike these scholars, we take our focus away from leaders and how leadership is (mis)performed. Instead, we join a stream of research in critical leadership studies in denaturalising the assumption that leadership, in any of the ways that it could be interpreted, is always essential and observable in any aspect of organisational life. This stream makes a case for ‘communityship’ (Mintzberg, 2009), arguing that a reliance only on leadership is potentially destructive, and contributes to intellectual and emotional deskilling organisationally and societally (Gemmill and Oakley, 1992). The illusion of invincibility and heroism embedded in leadership instigates a seductive process where the leader is located ‘above other men’ (Sinclair, 2007), undermining good decision-making, suppressing diversity (Tourish, 2013), and dampening energy and creativity (Raelin, 2016). Like Raelin (2016:149), we, instead, attempt ‘to bring the other back in’. We focus on the members of the community who use their own power to act outside of a leadership-followership rationale and reclaim what they have delegated (Mintzberg, 2009), a practice we call unleading. We do not take a for/against stance between leading and unleading as our main contention is that both are always present when organising, being more or less visible and practiced depending on organisational, social and individual circumstances.

It is not the first time researchers have challenged the sacredness of leadership and offered alternatives to bridge the separation between leaders and followers. Research on distributed leadership suggests leadership actors emerge anywhere in an organisation (Bolden, 2011). Unfortunately, when presented as a practice mitigating hierarchical power, distributed leadership becomes a way of rhetorically extending employees’ freedom of action while maintaining positional metaphors of hierarchy (Dainty et al., 2005). As Collinson and Collinson (2009) note, the assumption leadership emanates from an individual leader remains. Even the most egalitarian accounts relying on bottom-up, distributed leadership practices are co-opted by instrumental managerialism (Bendell et al., 2017) entailing a leader with the right and ability to empower others (Stacey, 2000), and are pseudo-empowering in that respect (Hatcher, 2005). What the pandemic has made visible is a redrawing of leadership practices where ‘the militant agent displaces the inactive agent’, bound to their communities ‘without requiring the community to strangle individualism’ (Grint, 2010: 103). Grint (2010) sensitised us to this possibility more than a decade ago and the pandemic gave us the opportunity to observe this phenomenon in action, which we coined unleading.

Unleading, in this sense, is a case for generative organisational theorising with its focus on process and practices (Langley, 2021). As Langley (2021: 252) defines in relation to the pandemic, ‘it is not the *effects* at a single point in time that are so interesting, but the temporally evolving processes they give rise to’. In this respect, unleading has become a sensitising concept in our intellectual inquiry (Blumer, 1954). While our practice-based focus does not lend itself to defining unleading under a single concept or analytical category, we relate our work conceptually with ‘leadership as a process’ (Knights and Willmott, 1992) and ‘leadership as practice’ (Raelin, 2011, 2016).

We suggest a multilocal and polycentric leadership that can emerge at any time as actors seek to make a new sense of the social. Informed by Smircich and Morgan’s (1982) ideas on the relational understanding of meaning-making, when individuals engaged in sensemaking processes in their everyday dialectical interactions during the sense-breaking COVID-19, unleading favoured collective sensemaking by ‘leadership actors’ (Fairhurst, 2008), providing a basis for action and shaping the reality for a larger community.

Diametrically opposed to the grand narratives of populism, Stacey (2010), with his work on complex responsive processes of relating, encourages us to pay attention to everyday practices and lived experience, to local conditions, to the quality of relationship, and to embrace rather than seek to resolve paradox and uncertainty. Certainly, these qualities, which Stacey (1996) termed as ‘extraordinary management’ were important in the pre-pandemic world. However, we contend that the pandemic amplified the ‘extraordinary’ impact of what was passed off as ‘ordinary’. The spontaneous actions and non-systematic initiatives, which already existed, but which were suppressed and wilfully overlooked in organisations and societies, have emerged from the shadows into the mainstream. Our empirical study aims to capture the learning from these actions and to reflect their potential to spawn new forms of leadership and followership that would reveal the potential of a ‘good enough’, instead of ‘total’ state of perfection. It is to the details of this study we turn our attention next.

## Data gathering and methodological choices

This study used discourse analytical research to scrutinise the tensions, paradoxes and surprises inherent in the spontaneous actions and non-systematic initiatives during the early stages of the pandemic and the effects they have on organising and our understanding of leadership. Recent research has emphasised the importance of discourse-analytical research in studying leadership storytelling and sensemaking (Schnurr and Schroeder, 2019) and interrogating leadership configurations and attributions in everyday relations and interactions (Holm and Fairhurst, 2018). However, we understood our methodological choices not as detached from, but embedded in, our experience of the pandemic, ‘living’ the news and opinion pieces and seeing them publicly disseminated through official and unofficial channels. This is akin to Hodgetts et al.’s (2007) conceptualisation of text-in-a-living-context with narratives of the media occurring in the social, understood as cultural practices – not only as texts – ‘capable of producing meanings and acceptance or resistance to these meanings’ (p. 383).

### Corpus building

The stories reporting on the public reactions to the pandemic as it unfolded in the newspapers, formed the corpus. To identify the corpus, we followed Neumann’s (2001, cited in Jensen, 2012) consideration of the reception of a discourse; its main points of reference are contained in a relatively limited number of texts. Using our cultural knowledge of the field in focus (Jensen, 2012) we identified three topics that created significant debate in the public and political space: Personal Protective Equipment (PPE), National Health Service (NHS) Charities Together providing support to staff in the UK’s NHS, and meals for key workers. They were instances of what Neumann (2001: 52, cited in Jensen, 2012) calls ‘monuments’ which act as discourse hubs or focal points.

We selected a sample of UK newspapers that represent quality, quality-like, and mass-market press; rightist and leftist political leanings; a presence in printed and online-only media. These newspapers were chosen because they represent different readerships, profiles and focus areas, which broadened the scope of the analysis. Table 1 summarises the sample.

**Table 1.** Newspapers included in the search sample.

			Political Leaning
**Quality Press**		*The Guardian*	Centre-left
		*The Telegraph*	Conservative
**Popular Press**	**Mass Market**	*Daily Mirror*	Centre-left
		*The Sun*	Conservative
	**Middle Market**	*i*	Centre-left
		*Daily Mail*	Conservative
**Subscriber Content**		*Financial Times*	Economically liberal
		*The Times, The Sunday Times*	Conservative
**Online Only**		*The Independent*	Centre-left

The dataset included newspaper articles retrieved from the *Nexis* database using the search strings: ‘NHS Charities Together’, Coronavirus AND (PPE OR masks OR visors OR gowns), and Meal AND ‘Key Workers’. The timeframe was set between 6^th^ March and 26^th^ April 2020, as the first death in the UK was reported in the newspapers on 6^th^ March whilst 26^th^ April was 2 weeks after the number of cases of peaked, for the first time, in the UK. The search string came after numerous explorative searches and readings, and the timeframe reflected the peaks and saturation points we identified by analysing trends in the reporting of stories on the topics.

### Downsampling and closer textual analysis

The search yielded 13,132 results. The list of results was reviewed using a random sampling strategy. The results were grouped in week-long increments and sorted by relevance. Then the top 30% of the articles in results list were reviewed. This process yielded 2,058 results which were included in closer textual analysis. Table 2 summarises the number of articles identified and included for further analysis in each stage of the review.

**Table 2.** The number of relevant news articles identified during each stage of the review.

		Included	Excluded	Justification for exclusion
**Database analysis**	PPE	12,200	—	
	NHS Charities	611	—	
	Meals	367	—	
*Total taken forward to next stage*		*13,132*		
**Title analysis**	PPE	2,150	10,050	Content excluded using a random sampling strategy^1^
	NHS Charities	611	0	—
	Meals	367	0	—
*Total taken forward to next stage*		*3,082*		
**Prelim. content analysis (pre-coding)**	PPE	92	2,058	Content was not directly relevant (e.g. reporting on PPE supply problems)
	NHS Charities	118	447	Content was not directly relevant (e.g. appeals to donate to NHS Charities Together)
	Meals	36	331	Content was not directly relevant (e.g. reporting implications of school closures on free school meals)
*Total taken forward to next stage*		*246*		
**Content analysis (post-coding)**	PPE	36	56	Content was either not directly relevant or insufficient detail to be meaningful
	NHS Charities	76	42	
	Meals	30	6	
***Total included in theory building***		*142*		

Table 3 captures the distribution of the final sample of articles according to news sources.

**Table 3.** News articles in the sample in respect of newspapers.

Source	PPE		NHS Charities		Meals	
—	No. of articles coded	No. coded data (mentions)	No. of articles coded	No. coded data (mentions)	No. of articles coded	No. coded data (mentions)
*The Guardian*	8	50	4	17	5	18
*The Telegraph*	10	45	8	52	9	37
*Daily Mirror*	0	0	3	17	1	13
*The Sun*	2	5	3	19	4	15
*i*	0	0	2	8	1	1
*Daily Mail*	4	18	15	43	3	6
*Financial Times*	1	4	0	0	0	0
*The Times, The Sunday Times*	3	8	0	0	3	0
*The Independent*	8	34	7	30	4	0

### Data analysis

We used an inductive approach to data analysis, termed *integrative methodology* (Fairhust and Putnam, 2019). Integrative methodology allowed us to combine elements of grounded theory with discourse analysis. Grounded theory generated a comprehensive understanding of incidents, drivers, intentions of actions undertaken while discourse analysis generated repertoires of speech, behaviours and arguments that signalled a change in how leading was talked about and practiced. In this respect, as a double-pronged methodology, this approach intensified levels of meaning in the data. Although grounded theory and discourse analysis are distinct methodologies, with their own supporting literature, both originate from a critical social constructivist commitment that enables their synthesis (Fairhust and Putnam, 2019).

The inductive nature of our approach meant that the analysis necessitated and benefited from an iterative selection and adoption of analysis steps that followed on from, but also informed, each other. In an attempt to shed light on the messiness of this process, we present our data analysis in five steps.

First, 246 news articles were read in detail, often more than once. Analysis was limited to press texts; images were not included. We omitted any parts of articles irrelevant to this study, for example, non-UK examples or examples not relevant to the three topics. Articles that did not include a rich portrayal of actors’ actions were also eliminated. The analysis then was action-specific and process-rich to allow us to hear the actors’ ‘voice’. We acknowledge that this voice was filtered down to us through mass media channels. However, given that we did not have the opportunity to obtain thick descriptions from the actors themselves through interviews, prioritising articles with extensive verbatim quotes from the actors was the closest we could get to their stories and explanations they provided to detail their context and motivations.

**Figure 1.**
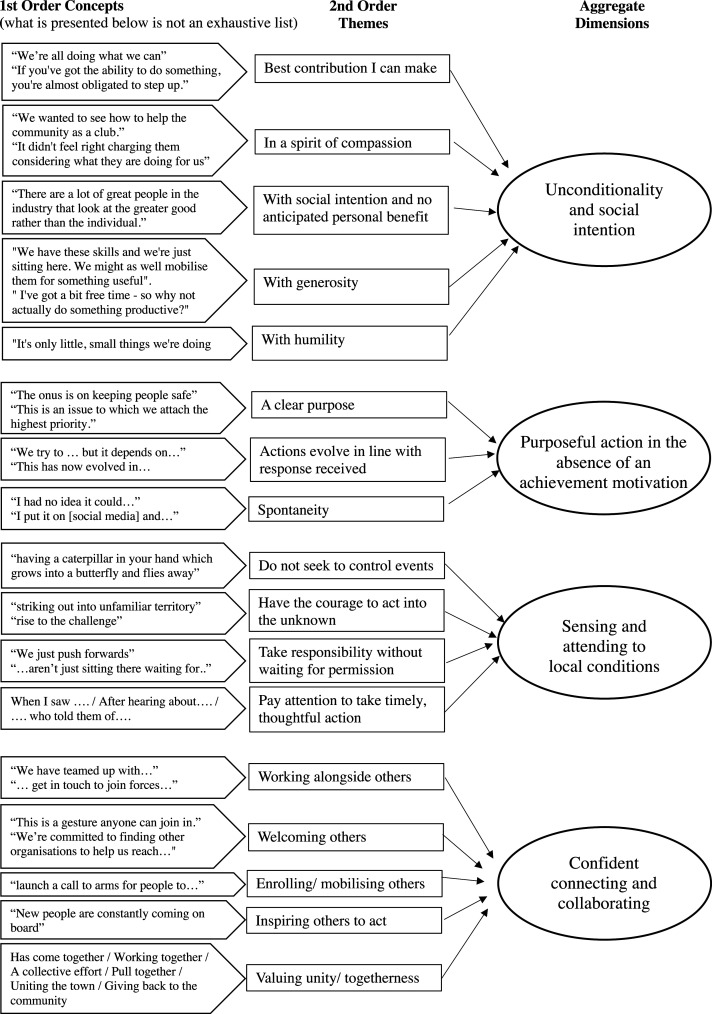
Data structure.

We then progressed onto a triple cycle of coding following Gioia et al. (2013), as illustrated in Figure 1.

In the initial coding, two of the authors individually scrutinised 142 news articles which are accounts of different people’s responses to the pandemic published in the news sources (captured in Table 2). The analysis began with an initial explorative line-by-line coding, selecting phrases or sometimes short paragraphs from these news articles as the first-order codes (left-hand column in Figure 1). The codes captured actions reported; critical incidents or drivers motivating actors to initiate their actions; their intentions; details of the relations they formed to implement and expand their actions; and accounts of how these relations were established and evolved. Most of these news articles also reported on how actors felt (e.g. proud, excited, surprised, humbled); these were also captured. To achieve inter-coder reliability, initially both authors coded the same articles, comparing codes, discussing, and resolving differences (Krippendorff, 2004). Intra-coder reliability was achieved through prolonged engagement with, and persistent observation of, the data (Lincoln and Guba, 1985), generating stable results when data segments were coded and recoded (Krippendorff, 2004).

In the second cycle, we engaged in axial coding (middle column in Figure 1), refining our selections, searching for relationships between and among first-order codes. We followed Madden’s (2010) ethnographic approach of thematic analysis, summarising, distilling and condensing data that resonated with our own reactions to the news. Sometimes we left the same codes created in the first cycle, others we assembled the codes into higher-order themes. Finally, in the third cycle, we looked for themes (right-hand column in Figure 1) to identify significant patterns of consistency and variation. This resulted in the identification of four overarching dimensions that comprise the basis of our emergent framework.

The critical consideration and identification of the properties and parameters of these themes, and associated speech and behaviour is where our approach overlapped with discourse analysis. In this fifth and final step, questioning the spoken, the assumed, the unspoken in the four overarching dimensions and in their practicing sensitised us to the dominant discourses and tensions with mainstream leadership rhetoric. During this step, we returned to the raw data which we examined against codes to examine discursive strategies and techniques, resolutions and effects. However, it is worth noting that even though we present a linear account of the analytical steps, in reality, this fifth step overlapped with the coding of grounded theory, as we went back and forth over the data and the codes. While we accept that the emergent framework is one of many potential interpretations (Van Maanen, 1998) of the uncontrolled and non-systematic actions during the pandemic, the integrative methodology allowed us to explore the socially constructed and socially constitutive character of the actors and their acts.

## Dimensions and elements of unleading: what is unleading and why does it matter?

**Figure 2.**
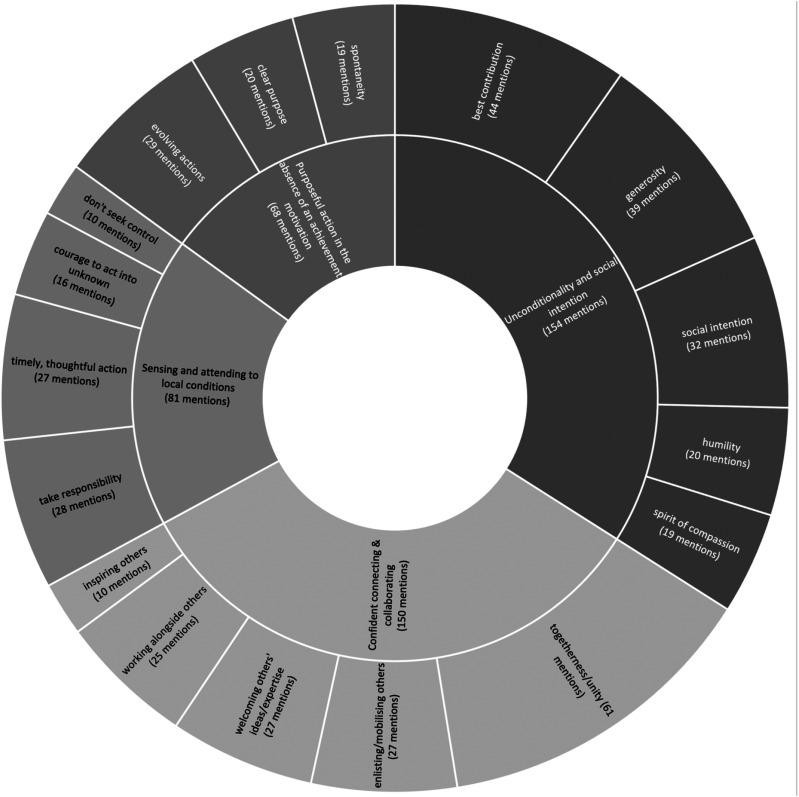
Dimensions and elements of unleadership.

Data analysis revealed that unleading under the pandemic is explained by four dimensions of actions: unconditionality and social intention; purposeful action in the absence of an achievement motivation; sensing and attending to local conditions; and confident connecting and collaborating. In total, coding of 142 articles revealed 453 occurrences of these dimensions, with secondary elements offering further nuance to the analysis. These are captured in Figure 2 and detailed below.

### Unconditionality and social intention

Unleading involves a proactive gesture that is deemed by the individual to be the *best contribution they can make to others and the social order*, in their current circumstances. These are gestures made with *humility*, with *generosity* and *in a spirit of compassion*. They are made *with social intention and without anticipated personal benefit.* When unleading, little attention is paid to the quantity, magnitude, and potential impact of gestures; it is a matter of doing whatever is possible ‘to help’ (April 4^th^, *The Sun*), with the intention ‘to carry on and help each other through’ (April 2^nd^, *Daily Telegraph*). The gesture can range from cooking 10 dishes a night for key workers in their home kitchens (April 13^th^, *The Guardian*), to retooling in an aerospace engineering firm to manufacture ventilator parts and supply them at cost because ‘it is the right thing to do’ (April 25^th^, *The Independent).*

Gestures may be adapted according to the response they call forth, as the story of Captain Sir Thomas Moore illustrates. Approaching his 100^th^ birthday, he set out with his walking frame to walk 100 laps of his garden, aiming to raise £1,000 for NHS Charities Together. Despite not knowing at the start ‘that his story would capture the hearts of the nation’ (April 15^th^, *Daily Telegraph*), when it did, he adapted: ‘as long as people believe that he’s worth investing in, he will keep walking’ (April 18^th^, *Daily Mail*). As a non-singer, he even recorded a single with Michael Ball to raise more funds, ultimately raising more than £38 million and received a knighthood for his contribution.

When unleading, feeling ‘compelled to help out’ (April 14^th^, *Daily Mail*) replaces skills and resources as the primary factor in deciding to act. For example, in Taunton, a group of 700 volunteers sewing scrubs and wash-bags for healthcare staff ranges from skilled sewers who are retirees to beginners who cannot sew. The oldest volunteer is 91 ‘who just wanted to do her bit…is adding buttons to all of the caps’ (April 23^rd^, *The Guardian*) as that was the best contribution she could make.

Whilst having the right skills and resource base may not be crucial, having the right intent is key; gestures are made with *social intention and without an anticipated personal benefit*. They constitute a pro-social behaviour that escapes traditional gift and counter-gift rules of exchange (Mauss, 1954). Aimed ‘at the greater good rather than the individual’ (March 25^th^, *The Telegraph*), unleading involves contributing whatever is possible – time, skills, resources – *with generosity* and often despite facing huge resource constraints. Examples from the news are plentiful; ranging from furloughed engineers offering their time and skills to 3D-print visors to companies donating medical grade plastic to support visor production; from film studios donating disposable gloves and suits to designers offering mask and visor design templates for free.

*Humility* also shines through in the discursive practicing of unleading. Two rhetorical devices used were: highlighting the mundanity and mediocrity of these acts in comparison to those of frontline healthcare professionals; and stressing that the act is born from having nothing better to do, yet wanting to act. So, while Tedros Adhanom Ghebreyesus, director-general of WHO tweets ‘This is such an inspiring story and extraordinary act of solidarity! Thank you so much Capt Tom Moore for such a wonderful idea and lesson of humanity!’ (April 17^th^, *Daily Mail*), Captain Tom plays down his gesture as ‘just a grandad doing some laps of his garden’ (April 18^th^, *Daily Mail*). The restaurant owner, who cooks 3,000 meals weekly for healthcare workers summarises his gesture as: ‘they’re the ones putting their lives at risk, all we are trying to do is give them something to eat’ (April 2^nd^, *The Telegraph*). The latter rhetorical device is illustrated by a London-based fashion designer who spearheads Fashion’s scrubs-making movement for the NHS: ‘We have these skills and we’re just sitting here. We might as well mobilise them for something useful’ (April 6^th^, *Daily Telegraph*).

Acting *with a spirit of compassion* drives, or contributes to, humility: ‘there are others having a much harder time’ (April 4^th^, *Daily Telegraph*). It is an issue of acting with social intention ‘just as a way of reaching those people who are alone at home and trying to show them that they don’t have to be lonely’ (April 10^th^, *The Independent)*.

### Purposeful action in the absence of an achievement motivation

Unleading implies an acceptance of uncertainty and ambiguity that facilitates dwelling in the unknown with no clear destination in view. Although there is a *clear purpose, spontaneity* replaces the ‘grand plan’ in fulfilling this purpose. Thus, *gestures and actions are adapted and evolve in line with the response they receive*.

The purpose of these acts and gestures is underpinned by social intent, such as ‘to keep as many individuals safe as they possibly can’ (April 6^th^, *Daily Telegraph*) or ‘to ensure those who need it most can have food delivered to their homes’ (1^st^ April, *Daily Mirror*). Unleading often starts with ‘a message out on the lads’ WhatsApp group’ (April 1^st^, *Daily Mirror*) or a post on Facebook or Instagram. Sometimes the actions are purely accidental as when a sales executive at a car dealer, saddened by not being able to attend his usual pub quiz, organised a virtual pub quiz with his friends through a Facebook event, attracting 30,000 attendees and raising £90,000 for the NHS (April 14^th^, *The Guardian*). The quiz has evolved since then and is now simulcasted on Facebook Live and YouTube raising money for the NHS and other charities.

This adaptive gesture-response cycle can also be seen in the café owner who started out providing a typical lunchbox with sandwich, snack, and drink, that evolved into a restaurant offering with vegan salads, flatbreads, pastries and fresh juices (March 25^th^, *The Telegraph*). Or an initial plan that quickly developed, required a newly formed group of 3D-printer owners to scale up to produce 1,000 visors per week (April 25^th^, *The Guardian*) and to think about nationwide production and supply channels. Many admit when they embarked on their action they had no idea what they could achieve, how it would be completed, where it would lead, and what resources could be tapped into. As a 13-year-old schoolboy who had been using his Christmas present to 3D-print visors put it: ‘it [is] like having a caterpillar in your hand which grows into a beautiful butterfly and flies away’ (April 25^th^, *The Guardian*).

Though these acts have a clear purpose, they are undertaken with no achievement motivation beyond making a difference. The *spontaneity* inherent in this tension between a clear sense of purpose and the absence of an achievement motivation allows timely, unconstrained acts into the unknown.

### Sensing and attending to local conditions

Unleading is not premeditated. There is no intention to *control events*. Instead, actions are agentic and responsive, undertaken *in a timely, thoughtful fashion* responding to the situation as it unfolds. By *paying, rather than seeking, attention* to local conditions, those who unlead act on the insight and information available to them now; they have the *courage to act into the unknown.*

When individuals notice ‘elderly neighbours struggle to buy the essentials they needed at the supermarkets’ (April 1^st^, *Daily Telegraph*), hear about ‘staff at the hospital [who] found themselves with nowhere to get food during long shifts’ (April 4^th^, *The Sun*) or simply see images on the internet ‘of medical staff showing the bruises and injuries caused by wearing masks during shifts’ (April 6^th^, *Daily Mail*), this prompts *taking timely, thoughtful action*. Thus, while Government waited and sought to centralise control of supply and distribution of PPE (Wilson, 2020), local initiatives emerged. These were often creative, for example, repurposing snorkel masks as ventilator masks for patients and making the design open source so others could do likewise (April 11^th^, *Daily Telegraph*). This proactiveness connotes *taking responsibility without waiting for permission*, or for authority to be delegated. News is replete with examples of companies dealing with hospitals, care homes, GP surgeries and pharmacies directly and working in response to their demands rather than waiting for the authorities.

*Rather than seeking to control events*, unleading focusses on controlling the quality of their gestures instead of controlling the response the gestures generate. A London-based fashion designer started with a message posted on her company’s Instagram account asking: ‘Can we make masks for you?’ Flooded with responses, she educated herself in making certified masks in sterile environments to gain the skills required to meet the need (April 4^th^, *Financial Times*).

As this example shows, unleading promotes *the courage to act into the unknown* and to admit to not knowing. Often the first step when unleading is taken with incomplete information and resources. For example, repurposing empty plastic kegs from a beer festival ‘to see if [he] could find another use for them, rather than seeing them be disposed of’ (April 10^th^, *The Independent*) resulted in the development of a design for masks. When initiating the action, a logistics manager for beer festivals, had neither a plan nor the necessary information and knowledge to put any prospective plan into action. Searching online for images of PPE, he ‘worked out how he could replicate it from recycled [kegs]’ before approaching local hospitals, care homes, chemists, and district nurses to provide them with masks for free (April 10^th^, *The Independent*).

Companies, as well as individuals, can incorporate these practices in their actions. For example, Royal Mint ‘decided to produce visors after searching online to see what medical equipment could be easily created on its site… On Wednesday at 9am [they] knew nothing about medical visors...within seven hours [the engineers] had created a medical visor, and within 48 hours it was approved for mass manufacture’ (March 28^th^, *The Independent*). As these examples show, an unleading attitude can include acting without future predictions or a calculation of whether the act is within the available skills, knowledge and resources.

### Confident connecting and collaborating

One of the defining aspects of unleading, is that actions are undertaken with no intention *to secure the commitment of others*. And whilst these actions may *inspire others to act*, and even succeed in framing the reality of others, they do not set out to do so. Unleading, however, involves answering openly to responders and connecting and collaborating confidently with them to achieve and go beyond their purpose. In doing so, they recognise the limits of their knowledge and resources and *willingly enrol and/or pass on responsibility* to better-placed others when these limits are reached. Some team up with other organisations and community initiatives. For example, a design and technology teacher recruited almost 20 colleagues from neighbouring schools to make PPE and attracted support from ‘Discover Islam, a grassroots organisation in Luton that facilitates and highlights the contributions of the Muslim community to wider society. It has…paid for almost all of the materials for the 7,500 visors produced at Chiltern Academy, as well as providing lunch every day for volunteers’ (April 23^rd^, *The Guardian*). Many others use crowdfunding appeals to secure the connections and resources they need to extend the impact of their acts when their initial resources are exhausted. The risk of appearing vulnerable by doing so is inconsequential.

Through connecting and collaborating confidently, unleading involves *acting in a community* to make a meaningful contribution: by feeling a real part of the community as they see the support of everyone coming together; bringing the community back together; managing the community spirit; and giving back to the community who have supported them.

With a profound sense of ‘communityship’ (Mintzberg, 2009) and ‘engaged social interaction’ (Raelin, 2016), instead of competing or seeking refuge in organisation, unleading might require joining forces with traditional rivals, as with the amateur rugby club delivering food to homes of NHS staff with ‘lads from rival clubs and football clubs’ to ‘show how [rugby] can bring people together for a good cause’ (April 1^st^, *The Daily Mirror*). The coming together may also form enduring new communities and collectives, as with Premier League footballers from rival clubs forming the #playerstogether collective to raise funds for NHS Charities Together that ‘could mark a sea change in the way top-flight top players deal with a range of important issues’ (April 19^th^, *Daily Mirror*).

Equally, many companies and organisations have collaborated with traditional rivals to innovate new products, services and supply chains, and have drawn creatively on the knowledge and experience of individuals throughout their organisation. In this sense, achieving the community’s common purpose, has greater significance than the individual or organisational agenda.

## Discussion

As predicted by Agamben (2005), at the time of writing of this paper, 9 months after the first lockdown began, with a new variant detected in the UK, the responses of those in formal leadership positions were based on a desire to remain in full, centralised control. Hancock, on December 21^st^, 2020, speaking on the BBC suggested that the new Coronavirus variant ‘is out of control and we need to bring it under control’ justified by a belief that a ‘state of perfection’ can only be achieved through the exercise of calculated, rationalised leadership control. In doing so, they have over-simplified, acting as though ‘all problems are puzzles rather than mysteries’ (Tourish 2020: 266).

What we demonstrated is that the (illusionary) full control is not the only response. Our findings shone a light on a series of uncontrolled initiatives that frequently pass unnoticed through a mainstream lens preoccupied by what leaders did or did not do as they responded to the COVID-19 crisis. We brought into view gestures of unleading, induced by the state of exception but appearing contradictory to a ‘state of perfection’ which leaders were seeking to achieve by maintaining and reinstating control. However, as anything that escapes the rhetoric of full control is considered dangerous under a state of exception, those unleading are at risk of being considered a menace. Reacting to fear and anxiety with spontaneity and originality, their apparently contradictory practices (e.g. enrolling and passing the mantle of responsibility to others when the limits of own resources are reached) could be taken as abnormalities and even, emulating Goffman (1963), expose them to stereotypification and social stigma. But as the risk of appearing vulnerable or seen as a failure are, for them, inconsequential, they are willing to accept that their actions may come to be seen as inappropriate or imperfect.

With unleading we find a resonance with Wabi-Sabi, and willingness to live with imperfection and not knowing. In contrast to the characterisation of ‘perfection’ and ‘imperfection’ as dichotomous conditions, the Japanese tradition of ‘Wabi-Sabi has no interest in permanence or completeness’, and therefore, ‘there is no need to lament about what could have been done to make something more perfect… imperfection of experience is what makes it so valuable’ (English, 2016: 8).

In accepting that actions may be incomplete or imperfect, those unleading may take timely, purpose-full action with no clear vision of where their actions will lead. This requires actors to act on the basis of information available to them, to spot ‘qualitative patterns’ that can be used to reason by analogy and intuition, rather than seeking certainty and a linear relationship between cause and effect (Stacey, 2000). And taking responsibility for their acts, they do not seek to – and recognise they cannot – control the response to them. Stacey (2010) describes this as a conversation between gestures and responses, where ‘gesture and response together constitute a social act in which meaning arises for both’ (p. 145). It is this conversation which allows adaptation and refinement of gestures by being alert to shifting, current conditions spotting and acting on the opportunity in collaboration with others.

When faced with a pandemic and the radical uncertainty that accompanies it, timeliness and insight into local conditions have particular significance; without them feedback loops can escalate rapidly (Stacey, 2000). For example, appropriate PPE for healthcare staff, primarily for their protection, also plays a role in reducing infection rates and making timely intervention of the essence. Thus, as our analysis of the practices of unleading and their impact suggests, complexity becomes subordinate to the capacity to act.

Wabi-Sabi with its attention to the imperfect and the beauty in the everyday and ordinary opens us up to a more nuanced understanding of the limitations of seeking only a state of perfection which justifies leadership as the only alternative when responding to exceptional circumstances. The dimensions of unleading highlight that under a state of exception, the social space does not necessarily become a totalitarian space explained through a univocal leader-follower rhetoric. Given the Foucauldian focus on the decentred and relational characteristics of power, latent in a network of relations (Torfing, 2013), the pandemic provoked an alternative multivocal space where others, not classified as followers or leaders, and who are usually invisible, were present.

Drawing on Starhawk’s (1987: 10–11) words from the indigenous tradition we see the ‘power-with’ – ‘the power to suggest and to be listened to, begin something and see it happen’ – allows individuals to craft more productive ways to exist. Focussing the attention and efforts away from trying to maintain ‘power-over’ (Starhawk, 1987: 10–11) the menaces through domination and control – what Raelin (2016:113) calls the ‘macro acts’ to mobilise central and sacred routines of authority – unleading’s ‘power with’ reframes the reality that is being observed and experienced by both individuals and society.

Unleading goes beyond actively distributing leadership practice and power, autonomy and agency up, down and across hierarchies. Whilst such distributed leadership practices are important in offering us a novel lens to observe the leader-follower relationship, their practice in the organisation is still dependent on the will of those in formal leadership positions to empower and entrust others and can be taken away at any time by the formal leaders. As characterised by Wainwright (2003: 193): ‘I participate, we participate, but they decide what kinds of issues we can decide’.

Unleading, in this respect, does not follow from a managerial initiative, but rather emerges spontaneously. Raelin (2016: 134) equally challenges the perception of leadership as a ‘dyadic relationship between leaders and followers’ and suggests a focus ‘to the activity of all those who are engaged, to their social interactions, and to their reflections and adjustments to their ongoing work’. With such a focus, unleading fosters collaborative agency and releases the agency of others. As Peck (1990) would frame it, rather than retreating from chaos into organisation, unleading promotes collaboration with and through others, embracing diversity without trying to heal or convert, in pursuit of true community.

Unleading, as a set of emancipatory, collaborative and agentic practices, has the potential to give voice to the ‘other’ to speak up in ways that are not manipulated, dampened or sanctioned by dominant individuals who have power over them (Raelin, 2016). Unleading then offers a complementary, not confrontational, redrawing of leadership dynamics in organisations. It pays attention to the micro decisions (spontaneous, uncontrolled, original) that are not explained by leadership/anti-leadership theories. It opens up a space more complex and flexible than the expected social space - usually represented by rigid bellicose archetypes of allies, enemies, rear and front lines and belligerent armies. Instead of representing the social under the pandemic as a war scenario and classifying the social actors as followers, leaders, traitors or loyalists, a flexible and multi-dimensional space emerges. In taking this learning into organisational life after the pandemic, we anticipate a flourishing of creativity and the humanising of our workplaces to accommodate the human spirit.

## Conclusion

The purpose of this paper has not been to disavow, disallow or challenge the role of leadership in the state of exception provoked by the pandemic. Rather we aim to make visible the reactions to the pandemic which cannot be explained through traditional leadership conceptualisations. We set out to trace how these reactions unfolded within this specific context to capture what was unique and meaningful about the phenomenon, to rethink and reimagine how we lead in organisations.

Our approach was similar to that of Gardiner (2016) who, inspired by Arendt’s work on human plurality, traced the ways in which authenticity unfolded within specific contexts with the argument that no leadership model would have offered the conceptual richness to account for the diversity of ways we live and lead. In a similar vein, we aim to encourage scholars and practitioners to expand their understanding of what it might mean to lead from our engagement with others and within organisational and social contexts, without fixating on leaders, their specific characteristics and behaviours and how these may or may not map on specific leadership models.

Joining scholars like Alvesson and Sveningsson (2003: 1456) in their commitment to move away from more ‘grandiose’ notions of leadership towards understanding the ‘leadership work’ in everyday situations, we appreciate the diverse ways of being (or leading) in the world. In doing so, we seek to capture the richness of ‘human plurality … within specific relational contexts at different moments in time’ (Gardiner, 2016: 633). We treat the pandemic as an opportunity for generative theorising (Langley, 2021) – an occasion for sensemaking but also an occasion for sensegiving to the responses people gave following societal concerns, such as the PPE shortage. The analysis of practices of unleading, which were spontaneous and improvised by their actants, shows a complex discursive structure, far removed from an aleatory succession of disintegrated narratives without connection. Behind the apparently disconnected and anecdotal news, there emerges a network of people doing things for others within a consistent four-dimensional discursive space, with clearly defined elements.

We wrote this paper in the midst of the pandemic. As Raelin (2016: 134) claims ‘our knowing, then, is in sync with the reality we are experiencing as we interact with the environment’. We are conscious that we are writing in the ‘living present’ as the virus is still among us, living the state of exception every day, where meaning emerges from the interaction of the past and the future (gesture and response, respectively) (Stacey, 2010), whilst simultaneously analysing reactions and learning from the experience. Unleading, its meaning and our knowing are, in this sense, fluid and it would take more space than this paper allows to analyse the evidence of the full fluidity and intermingling of these elements.

As our understanding of unleading evolves, we may reveal its different faces. The context within which our intellectual inquiry emerged, resulted in a dataset that caught the imagination of people at the time, bringing ‘hope in the dark’ as Solnit (2005) might describe it. The data, in that respect, had a romantic appeal – the very same appeal that critical leadership scholars have been fighting against since Meindl et al.’s (1985) classic article on the romance of leadership. Meindl and colleagues contended that there was a tendency to attribute excessive credit or blame to leaders for organisational successes (or failures). With our analysis we do not intend to contribute to what Kempster and Carroll (2016: 8) refer as ‘a new romanticism’, excessively crediting the actors in our stories for the post-pandemic recovery. We acknowledge that the impact of these actions and associated practices were shaped by properties other than the actors undertaking them.

As much as we tried to ascribe greater importance to the practices than to specific people and their persona, the positivity inherent in our dataset has resulted in a unitarist, uncontested representation of unleading. We recognise that the four dimensions that emerged from our analysis and associated practices, which in this instance contributed to the social good, in a different context could have had markedly different, possibly destructive, effects. We also recognise that concepts such as ‘social good’, ‘doing good’ and ‘the best contribution’ do not have universally accepted meanings and can be defined in multiple contested ways according to various agendas. Further research into unleading in different contexts might help us explore its darker side.

Furthermore, we used published data and, in a sense, created a sample of acts of unleading as recognised in the mainstream media, despite setting our focus on uncontrolled initiatives which typically go unrecognised through a mainstream lens. Undoubtedly, there were other unleading initiatives equally and even more remarkable which did not end-up in our sample as they, for whatever reason, did not catch media attention. Even though this limitation does not affect the validity of our findings, collecting data on other initiatives from alternative media or through ethnographic research in community groups would have led to a richer dataset. Future research analysing social networks, potentially through discourse analysis of netnographic interactions could illuminate other ways of discursively constructing unleading.

We are aware that from a managerialist angle it requires a dramatic leap of faith to believe that unleading can be performed in organisations. But what might we learn from unleading when managing and organising? In liberating social practices from the usual narratives of control, infantilisation, patronisation and demonisation of others constructed as enemies, those unleading have demonstrated their creativity and resourcefulness in collaborating to work around the barriers created by centralised control, exposing the limitations of strong leaders who cling determinedly to them. From the perspective of organisational leaders, learning from unleading involves a number of twists of attention, for example: from anticipated results to the quality of my contribution; from good processes to making good judgements; from the pursuit of a state of perfection to ‘good enough’; and from the leader-follower dyad to collaborative agency. We may then liberate the potential of unleading as a practice to stimulate, encourage and promote within the organisational space. We can then imagine the emergence of a self-confident society, not promoting strong leaders but instead seeking leadership from everywhere and nowhere at a collective and individual level, the spontaneous reaction of individuals moved by compassion and generosity and not by the diktat of someone else. Perhaps the most important lesson from unleading then is that it is within all our grasps.

## Note

1. The list of results was reviewed using a random sampling strategy. Specifically, the results were grouped in week-long increments, and for each week, the top 30% of the articles in the list of results, which were sorted by relevance, were reviewed. This process yielded 2058 results which were included in Stage 2 - Title Analysis.
